# Estimation of Prestress Force Distribution in Multi-Strand System of Prestressed Concrete Structures Using Field Data Measured by Electromagnetic Sensor

**DOI:** 10.3390/s16081317

**Published:** 2016-08-18

**Authors:** Keunhee Cho, Jeong-Rae Cho, Sung Tae Kim, Sung Yong Park, Young-Jin Kim, Young-Hwan Park

**Affiliations:** Structural Engineering Research Institute, Korea Institute of Civil Engineering and Building Technology, 283 Goyangdae-Ro, Ilsanseo-Gu, Goyang-Si, Gyeonggi-Do 411-712, Korea; kcho@kict.re.kr (K.C.); chojr@kict.re.kr (J.-R.C.); esper009@kict.re.kr (S.T.K.); sypark@kict.re.kr (S.Y.P.); yjkim@kict.re.kr (Y.-J.K.)

**Keywords:** optical fiber sensor, electromagnetic sensor, prestress, smart strand, multi-strand system

## Abstract

The recently developed smart strand can be used to measure the prestress force in the prestressed concrete (PSC) structure from the construction stage to the in-service stage. The higher cost of the smart strand compared to the conventional strand renders it unaffordable to replace all the strands by smart strands, and results in the application of only a limited number of smart strands in the PSC structure. However, the prestress forces developed in the strands of the multi-strand system frequently adopted in PSC structures differ from each other, which means that the prestress force in the multi-strand system cannot be obtained by simple proportional scaling using the measurement of the smart strand. Therefore, this study examines the prestress force distribution in the multi-strand system to find the correlation between the prestress force measured by the smart strand and the prestress force distribution in the multi-strand system. To that goal, the prestress force distribution was measured using electromagnetic sensors for various factors of the multi-strand system adopted on site in the fabrication of actual PSC girders. The results verified the possibility to assume normal distribution for the prestress force distribution per anchor head, and a method computing the mean and standard deviation defining the normal distribution is proposed. This paper presents a meaningful finding by proposing an estimation method of the prestress force based upon field-measured data of the prestress force distribution in the multi-strand system of actual PSC structures.

## 1. Introduction

**Figure 1:**
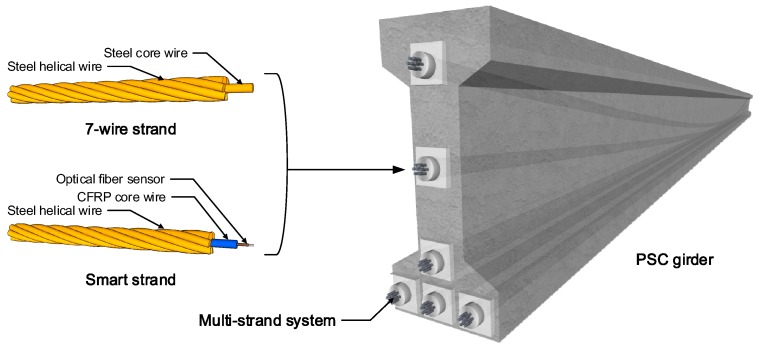
Prestressed concrete (PSC) structure with multi-strand system.

Prestressed concrete (PSC) allows the structure to overcome concrete’s natural weakness in tension by the introduction of a compressive force. This prestress force in the PSC structure is generally introduced by means of steel strand. The recently developed smart strand can be used to measure the prestress force in the PSC structure from the construction stage to the in-service stage. The smart strand imparts the strand with measuring capability, achieved by replacing the core wire by a steel tube [1], a carbon fiber reinforced polymer (CFRP) rod [2], or a glass fiber reinforced polymer (GFRP) rod [3] in which a fiber optic sensor is embedded. Figure 1 depicts a PSC girder prestressed by multi-strand systems composed of a traditional 7-wire strand and smart strand.

The higher cost of the smart strand compared to that of the conventional strand renders it unaffordable to replace all the strands by smart strands and results in the application of only a limited number of smart strands in the PSC structure. However, the prestress forces developed in the strands of actual PSC structures differ from each other [4], which means that the prestress force in the multi-strand system cannot be obtained by simple proportional scaling of the measurements provided by a limited number of smart strands. Consequently, there is a need for a rational method enabling correlation of the measurement of the smart strand and the overall prestress force distribution.

Cho et al. [5] observed the distribution pattern of the prestress force in the anchor heads in a series of jacking tests on a specimen 20 m in length, and proposed a method to relate this distribution of the prestress force in the multi-strand system to the measurement of the smart strand. This study was the first one to systematically deal with the distribution of the prestress force in the anchor head, but exploited a limited amount of data acquired from an experimental specimen, not field-measured data from actual structures. 

Accordingly, the present study intends to derive realistic and reliable patterns of the prestress force distribution per anchor head by means of a number of data on various factors of the multi-strand system. These data were measured on site by electromagnetic (EM) sensors, and the resulting distribution patterns of the prestress force were observed to adhere to an adequately fitting probabilistic distribution. Moreover, this study also intends to propose a method enabling correlation of this distribution with the smart strand.

## 2. Measurement

### 2.1. Measurement Setup

The distribution of the prestress force in the tendon in the PSC structure can vary according to the length and curvature of the tendon, following the effect of the friction of the tendon. Since the tendons may be intertwisted, the distribution can also be influenced by the number of tendons and the level of prestress. Moreover, apart from these factors, the distribution of the prestress force in the tendon is also subordinate to the skill and practice of the technician. However, the latter factors relative to the technician cannot be quantified. Accordingly, the evaluation of the extent of the influence on the distribution of the prestress force was done only for the factors that can be quantified.

**Figure 2:**
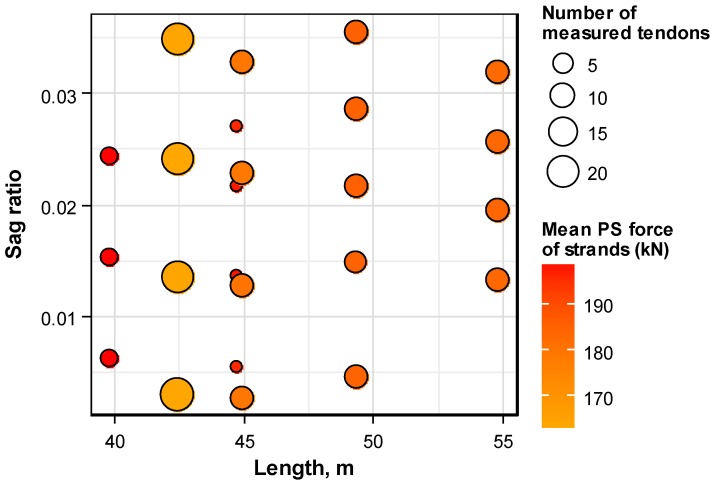
Distribution of considered PSC structures per length and sag ratio.

The considered PSC structures were thus selected to include a range of structures actually used on site, and to comprehensively reflect these influencing factors in order to derive realistic and rational distribution patterns of the prestress force. Table 1 lists the PSC structures selected for measurement, considering these aspects. The lengths of the girders range between 39.5 m and 54.8 m, the tendon sag ratios vary from 0.0112 to 0.1420, and the number of strands per anchor head runs between 13 and 16; these measured details include most of the commonly used prestressing factors of the multi-strand systems. Figure 2 plots the diagram that concurrently indicates the distribution of the girder length, tendon sag ratio, level of prestressing, and number of measured tendons for the structures considered in this study. It appears that the listed PSC structures achieve even distribution with regard to the girder length and tendon sag ratio. The prestressing levels per strand are between 164 kN and 200 kN, which includes the range actually found in real structures. In terms of numbers, 49 girders, 194 tendons, and 2761 strands were measured.

### 2.2. Measurement Method

A number of sensors equal to the number of strands were used to measure the distribution of the prestress force of each individual strand in the tendon. In addition, it seemed advisable to adopt reusable and cheap sensors to collect large volumes of data. The use of contact sensors, like the load cell, to measure the prestress force would require them to be installed between the concrete structure and the anchor. Such disposition would render it impossible to reclaim the sensor after completion of prestressing, making contact sensors inappropriate for this study where the acquisition of a huge amount of data is necessary. Besides, the contactless electromagnetic (EM) sensor can be installed between the anchor head and the hydraulic jack. This enables not only the measurement of the prestress force, but also reuse of the sensor, which is in alignment with the purpose of this study. Accordingly, the EM sensor was selected for the measurement of the prestress force in individual strands.

The EM sensor is a contactless sensor that was conceived based on the fact that the induced magnetic flux and current passing through the sensor experiences changes according to the stress state of the material [6]. This sensor is used to measure the resisting load in the tendons of PSC structures or the stay cables of cable-stayed bridges [7,8].

**Figure 3:**
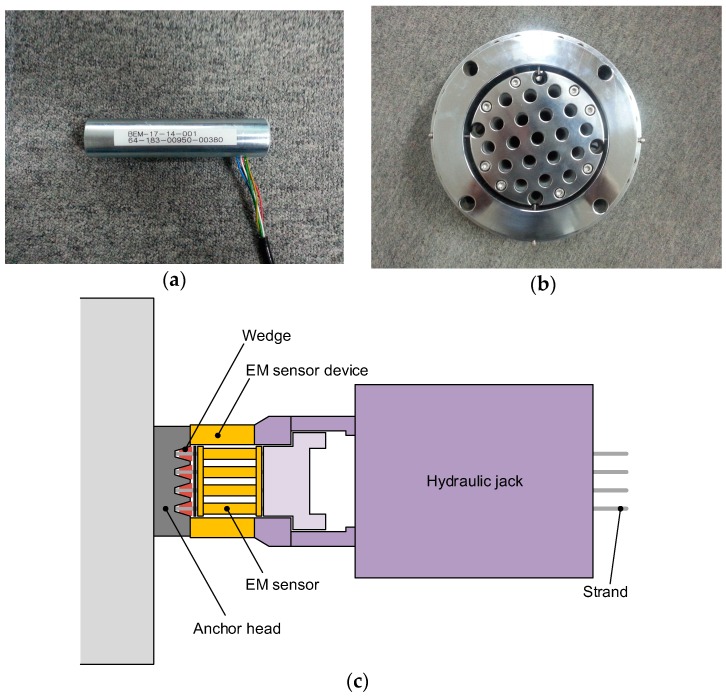
Developed device: (**a**) Electromagnetic (EM) sensor; (**b**) EM sensor device; (**c**) Schematic drawing of the composition of sensing device for the measurement of the prestress force distribution (EM sensor device + hydraulic jack).

In this study, a special device (hereafter referred to as EM sensor device) was conceived to deposit EM sensors onto each strand so as to measure the prestress force in each individual strand of the multi-strand system. The EM sensor was fabricated to fit with the dimensions of the strands and anchor heads and was calibrated with respect to the strands used in each considered site. This EM sensor device was used to measure the prestress force in each strand by attaching it to the head of the hydraulic jack (Figure 3). The measured prestress force was thus the jacking force between the anchor head and the hydraulic jack.

**Figure 4:**
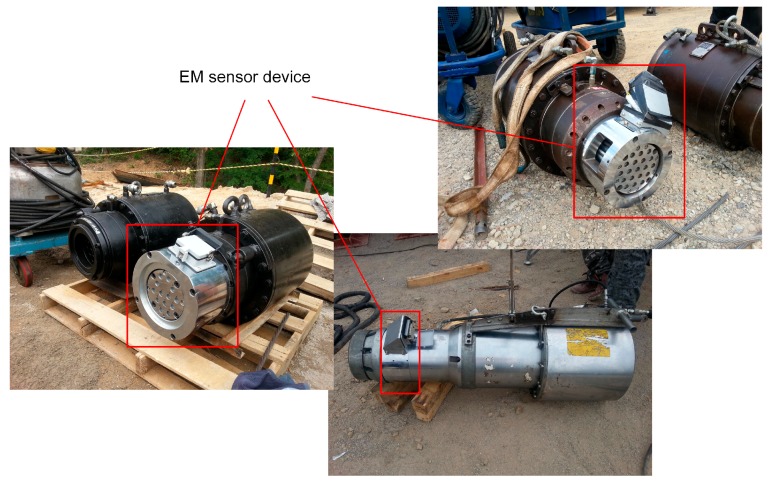
EM sensor device attached to hydraulic jack.

**Figure 5:**
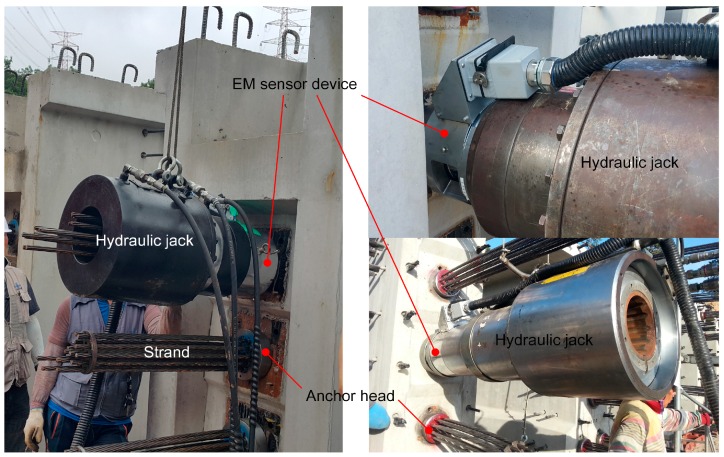
Prestressing and measurement on construction sites.

Since the EM sensor device must be attached to the hydraulic jack, the device was fabricated to be adaptable to the various jacks actually used on site. Table 2 lists the three hydraulic jacks considered in this study. Figure 4 shows photographs of the EM sensor device attached to the hydraulic jack. The EM sensor device was used to conduct measurements on PSC girder construction sites as shown in Figure 5.

## 3. Results and Discussions

### 3.1. Distribution of Measured Prestress Force

The last two columns of Table 1 arrange the mean of the mean prestress forces and the mean of the standard deviation of the prestress forces per anchor head by classifying the whole sets of measured data with respect to the girder length and tendon sag ratio. The distributions of the mean prestress force per anchor head have means ranging between 164 kN and 200 kN, and standard deviations ranging between 5.1 kN and 8.4 kN.

**Figure 6:**
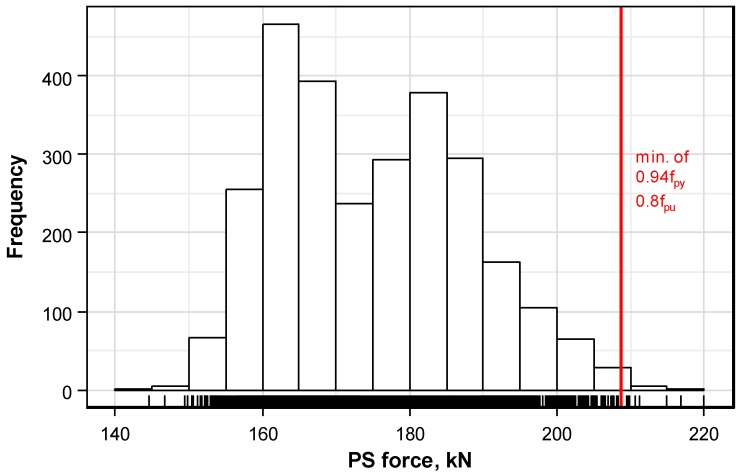
Frequency histogram of prestress force measured in considered strands.

Figure 6 plots the frequency histogram of the prestress force distribution measured on the whole set of strands. The prestress force per strand ranging between 144 kN and 220 kN is seen to cover a wider range than the mean of the prestress force per anchor head.

ACI 318-14 [9] limits the maximum tensile stress of the tendon due to jacking force during prestressing to a value below $0.94f_{py}$ (where $f_{py}$ = yield strength), $0.80f_{pu}\;$(where $f_{pu}$ = ultimate strength), and a proposed value by the manufacturer. With a yield strength of 234.6 kN and ultimate strength 261 kN for a Grade 270 (1860) strand with a diameter of 15.2 mm, as specified in ASTM 416/416M [10], gives a maximum limit stress of 208.8 kN for the strand. However, as shown in Figure 6, some strands exceed this stress limitation. Even if the overall average of the mean prestress force per anchor head does not exceed the criterion, the prestress force of some individual strands exceeds the limit stress criterion. Accordingly, it is necessary to limit the mean prestress force to a level that prevents the exceedance of the maximum limit per anchor head to solve this problem.

Since the histogram of the prestress force shown in Figure 6 may exhibit various distributions according to the level of the design prestress force, the prestress force per anchor head is decomposed as follows in order to exclude the influence of the design prestress force.

(1) $$f_{i}=f_{m}+f_{r,i}$$

where $f_{i}$, the prestress force of *i*th strand in the anchor, is expressed as the sum of the mean prestress force per anchor head, $f_{m}$, and the residual prestress force of the strand, $f_{r,i}$.

**Figure 7:**
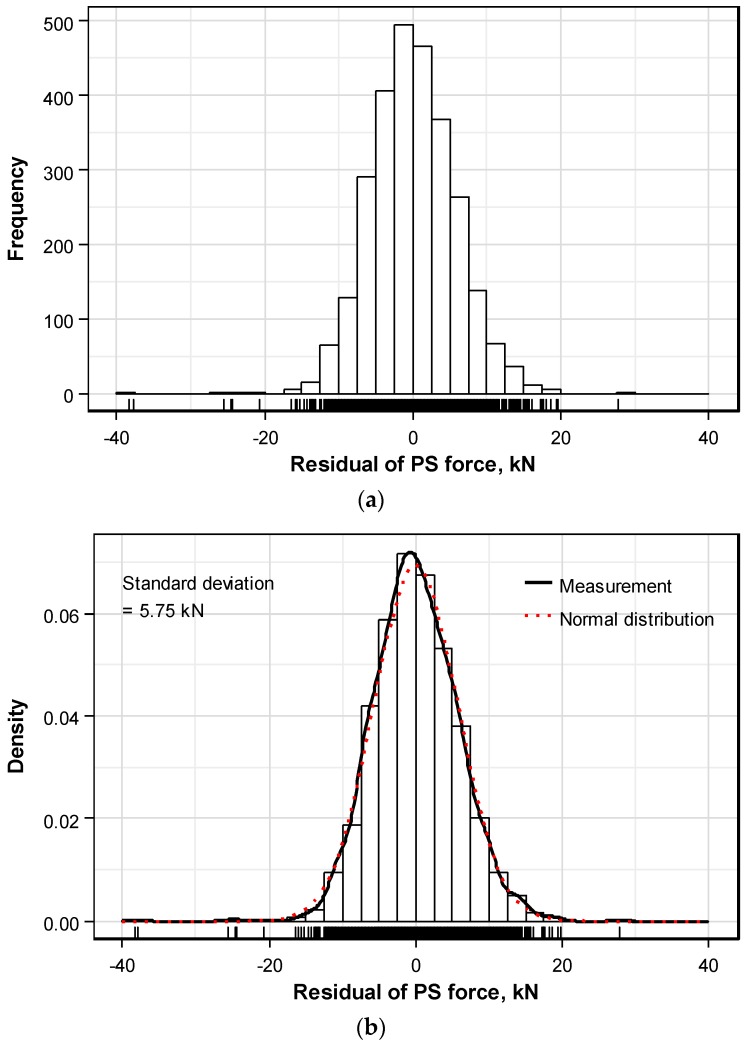
Distribution of residual prestress force excluding the mean prestress force from the prestress force of the strand: (**a**) Frequency distribution; (**b**) Density distribution.

Figure 7 redraws Figure 6 in term of the residual prestress force from which the influence of the mean prestress force is removed. Figure 7a represents the frequency distribution and Figure 7b compares the density distribution and normal distribution, where the normal distribution is drawn for a mean of 0 kN and standard deviation of 5.75 kN of the residual prestress force. Since both density distribution and normal distribution are in good agreement, the distribution of the measured prestress force can be assumed to follow the normal distribution. However, this assumption relates to the distribution of the prestress force of individual strands and does not mean that the distribution of the prestress force per anchor head is normal.

### 3.2. Test for Normality

The previous section dealt with the distribution pattern of the prestress force relative to the whole set of the 2761 measured strands. On the other hand, the present section intends to examine the distribution pattern of the prestress force by limiting the scope to the anchor head. First, normality test is conducted to determine if the distribution pattern of the prestress force per anchor head follows the normal distribution for the whole set of anchors.

The number of strands per anchor runs around 13–16, which constitutes a relatively small number of samples. As a matter of fact, it is rather difficult to test accurately the normality of a distribution using a small number of samples. Therefore, the Shapiro-Wilk test [11] is selected here for the normality check owing to the relatively high accuracy provided by this method for a small number of samples [12]. 

**Figure 8:**
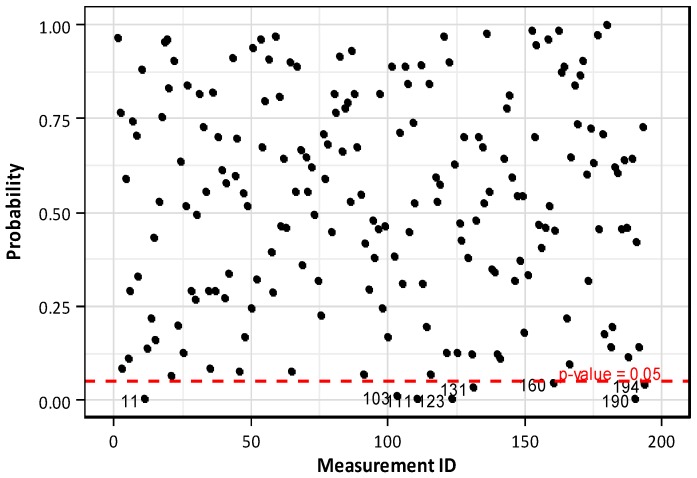
Normality test of residual prestress force per anchor head.

Figure 8 plots the normality test results relative to the 194 anchors considered in this study. It appears that only 8 data have significance probability lower than 5% while the other data present significance probability higher than 5%. Since only a very small portion of the data failed the normality test, it can be assumed reasonably that the distribution of the prestress force per anchor head follows the normal distribution.

**Figure 9:**
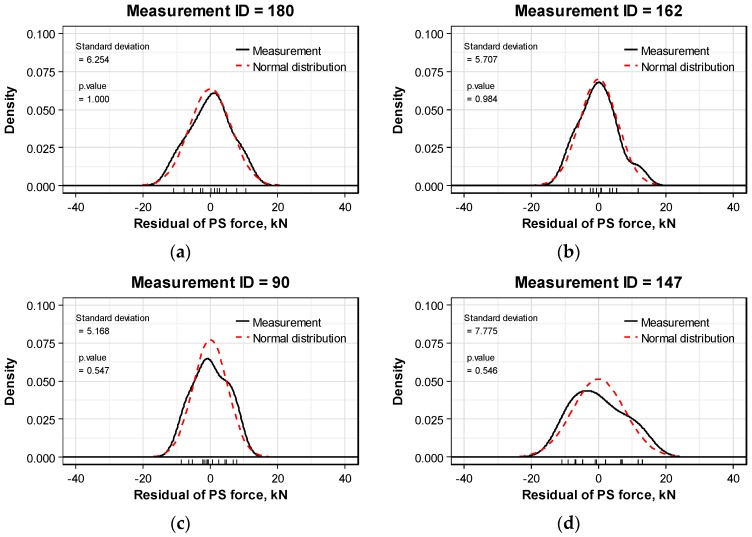

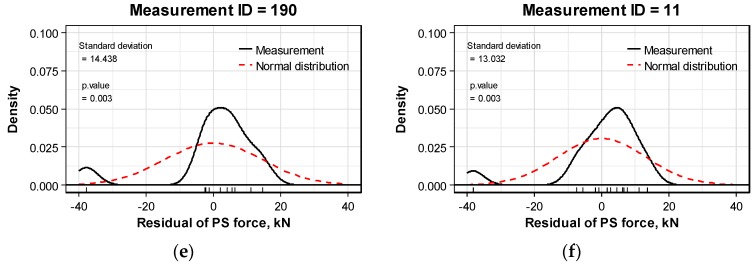
Comparison of the distribution of prestress force per anchor head and the normal distribution: (**a**,**b**) Examples of good agreement with the normal distribution; (**c**,**d**) Examples of medium agreement with the normal distribution; (**e**,**f**) Examples of disagreement with the normal distribution.

Two sets of data with high significance probability, two sets of data with medium significance probability, and two sets of data with low significance probability were selected to check the extent of agreement of the actual distribution with the normal distribution. The results plotted in Figure 9 show good agreement for the data with high significance probability and clear disagreement for the data with low significance probability.

### 3.3. Evaluation of Factors Influencing the Standard Deviation of the Prestress Force per Anchor Head

The previous section validated the assumption that the prestress force distribution of the multi-strand system follows the normal distribution. What is now needed is the mean and the standard deviation to define the normal distribution. The mean can be easily obtained by dividing the prestress force indicated by the hydraulic jack by the number of strands inserted in the anchor. Therefore, the distribution of the prestress force in the anchor can be defined if the standard deviation is obtained. To that goal, this section examines the distribution pattern of the standard deviation per anchor head.

**Figure 10:**
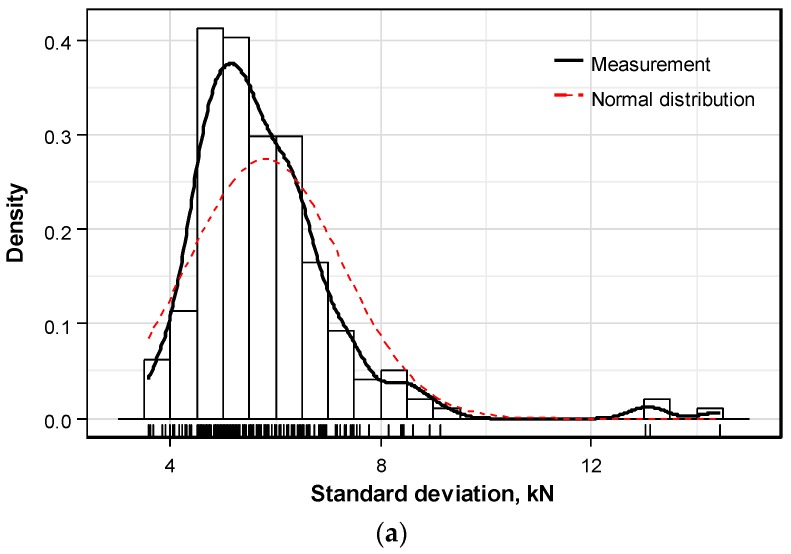

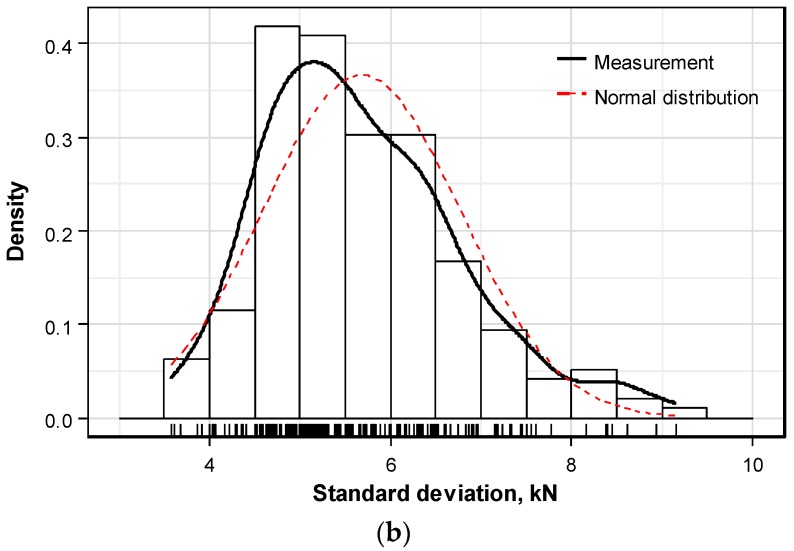
Distribution of standard deviation of prestress force per anchor head: (**a**) Whole set of measured data; (**b**) Measured of data excluding outliers.

Figure 10 plots the density histograms of the standard deviation per anchor head. Figure 10a relates the distribution for the whole set of data and Figure 10b is drawn when the 3 outlier data sets are excluded from the considered data. It appears that the distribution of the standard deviation for the whole set of data, including the outliers, has mean of 5.80 kN and standard deviation of 1.45 kN, whereas the same distribution for the data excluding the outliers has mean of 5.67 kN and standard deviation of 1.09 kN. This indicates that large distortion is generated by the 3 outliers. As shown in Figure 9c, these 3 outliers correspond to the computation of a large standard deviation caused by the loose arrangement of some strands in the tendon. However, such misplacing of a part of the strands can be attributed to careless prestressing work and should not occur in cases of appropriate prestressing. Accordingly, these 3 outliers are discarded from the subsequent analysis. 

Consequently, compared to the normal distribution, the distribution of the standard deviation of the prestress force per anchor head excluding the outliers appears to be slightly skewed to the left and presents a long tail on the right.

The tendency of the distribution of standard deviations of prestress force per anchor head is now examined according to the change in the length of the structure, the curvature of the tendon, and the mean prestress force. 

**Figure 11:**
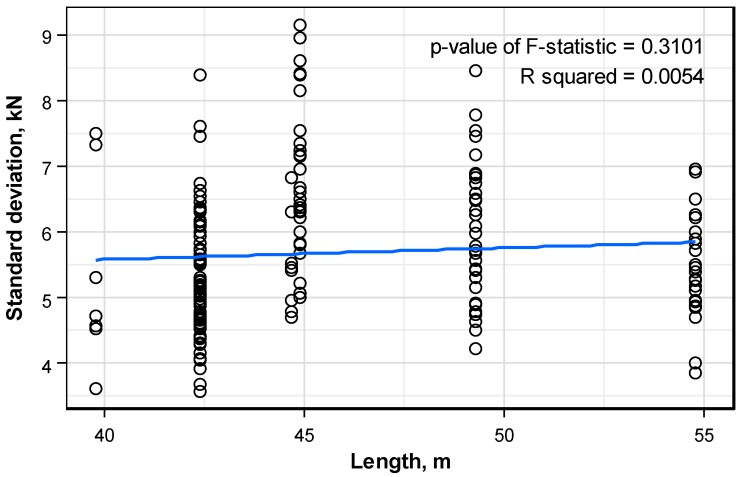
Distribution pattern of the standard deviation of the prestress force per anchor head according to the structure length.

Figure 11 plots the distribution pattern of the standard deviation of the prestress force per anchor head with respect to the length of the structure. The dots indicate the distribution of the standard deviation and the solid line figures the linear trend line. The pattern of the distribution of the standard deviation does not show particular tendency according to the increase of the structure length. In addition, the p-value of the F-statistic indicating the validity of the regressive line is smaller than 0.05 and shows that the regressive line is not valid. Therefore, it can be concluded that the length of the structure has practically no effect on the standard deviation.

**Figure 12:**
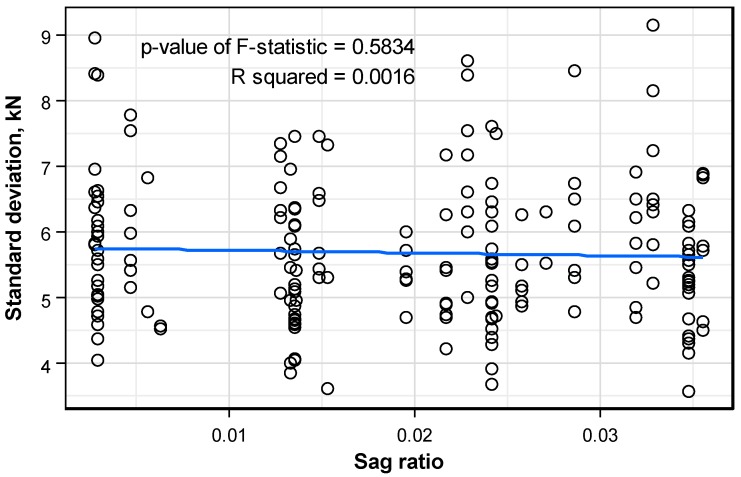
Distribution pattern of the standard deviation of the prestress force per anchor head according to the tendon sag ratio.

Figure 12 plots the distribution pattern of the standard deviation of the prestress force per anchor head with respect to the sag ratio of the tendon. In this case, the standard deviation seems to reduce with larger sag ratio, but very slightly and without clear trend. Moreover, the regressive line appears to be invalid since the p-value of F-statistic is smaller than 0.05. Accordingly, similar to the structure length, it can also be concluded that the tendon sag ratio has practically no effect on the standard deviation. 

**Figure 13:**
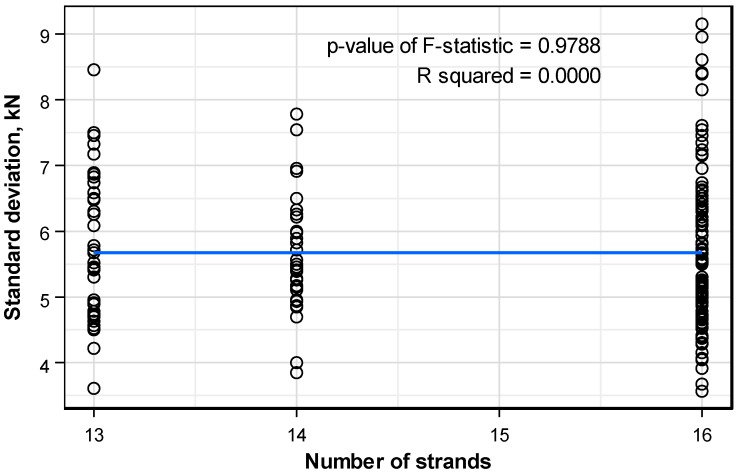
Distribution pattern of the standard deviation of the prestress force per anchor head according to the number of strands per anchor.

Figure 13 plots the distribution pattern of the standard deviation of the prestress force per anchor head with respect to the number of strands per anchor. Here also, it is also concluded that the number of strands per anchor has practically no effect on the standard deviation.

**Figure 14:**
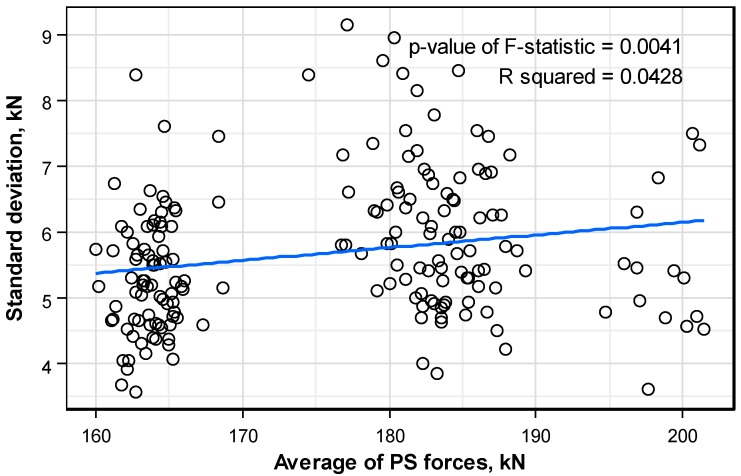
Distribution pattern of the standard deviation of the prestress force per anchor head according to the mean prestress force.

Figure 14 plots the distribution pattern of the standard deviation of the prestress force per anchor with respect to the level of prestress per anchor. Unlike the precedent factors, the level of the prestress force appears to influence the distribution of the standard deviation of the prestress force per anchor head. In addition, the p-value of F-statistic indicates the validity of the regressive line with a value smaller than 0.05. Concretely, higher level of prestress tends to gradually increase the standard deviation of the prestress force. However, the R squared relative to the regressive line indicates poor suitability with a value of about 0.04. This means that the unquantifiable skill and practice of the technician has a larger influence on the standard deviation relative to the distribution of the prestress force per anchor than the factors considered in this study.

### 3.4. Derivation of Formula for the Estimation of the Prestress Force Distribution

The evaluation of factors influencing the standard deviation of the prestress force per anchor head revealed the linear proportionality between the level of prestress and the standard deviation, and the quasi-absence of effect on the standard deviation of the structure length, tendon sag ratio, and number of strands. Accordingly, the standard deviation of the prestress force per anchor head can be formulated by a linear equation involving the level of prestress per anchor head from Figure 14 and the trend line can be expressed as follows.

(2) $$\sigma =0.019f_{m}+2.286\;\left[\text{kN}\right]$$

Consequently, the normal distribution $N\left(f_{m},\;\sigma^{2}\right)$ of the prestress force per anchor head can be defined using mean prestress force per anchor head $f_{m}$, and the standard deviation σ obtained using Equation (2).

### 3.5. Tracking of Change in Prestress Force Distribution

The pattern of the distribution of the prestress force per anchor head could be estimated based upon the observation of the measured data. This section intends to find a solution that enables tracking of the distribution of the prestress force in an anchor using the measurement of the smart strand when one strand among those of the anchor is replaced by the smart strand.

**Figure 15:**
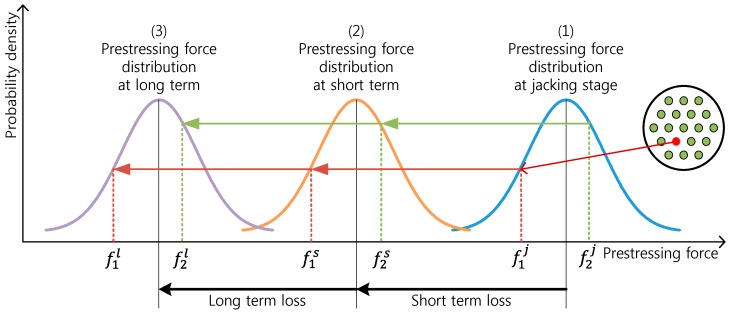
Concept of the estimation of the prestress force distribution.

During the prestressing of an anchor, the overall prestress force can be known by means of the load cell of the hydraulic jack. The mean prestress force can thus be obtained by dividing this total prestress force by the number of strands inserted to this anchor, and the standard deviation can then be calculated using Equation (2). Using these values, the distribution of the prestress force in the anchor can be estimated as a $N\left(f_{m},\sigma^{2}\right)$ normal distribution. This part corresponds to the first step in the estimation of the prestress force distribution shown in Figure 15. Here, the measurement given by the smart strand corresponds to just one value in the distribution. In such a case, this measurement may not be in agreement with the mean prestress force.

After prestressing, the distribution pattern of the prestress force experiences continuous change due to the occurrence of long-term and short-term losses of prestress according to the use of the PSC structure. The long-term loss and the short-term loss of prestress corresponding to steps (2) and (3) in Figure 15 result in the variation of the value measured by the smart strand, which can be used to track the change in the state of the distribution of the prestress force. 

Considering that the prestress force measured by the smart strand has changed by Δf from the prestressing stage to a definite time, it is reasonable to assume that the distribution curve of the prestress force has also translated by Δf during that period. Even if the change in the distribution of the prestress force can be computed more accurately considering the deformation inside the section of the structure, increasing the accuracy is not worth the effort since the distribution pattern of the prestress force is approximated as a normal distribution. Accordingly, the changed mean prestress force at the considered time becomes $f_{m}+\Delta f$. Noting that the standard deviation has not changed, this means that the distribution of the prestress force at this time can be estimated by the $N\left(f_{m}+\Delta f,\;\sigma^{2}\right)$ normal distribution [5].

## 4. Conclusions

This study verified that the individual strands pertaining to the same anchor could sustain different prestress forces and proposed a method enabling the estimation of the distribution of the prestress force in the anchor head in order to improve the applicability of the smart strands when present in limited number in PSC structures. To that goal, a special electromagnetic (EM) sensor device, adaptable to various hydraulic jacks, was fabricated to measure the prestress force of each individual strand in the anchor head. This device was used to measure the distribution of the prestress force in a construction site having real PSC girders. Field-measurement of the prestress force was conducted on a total of 24 types of structures with different levels of prestress, number of strands, curvature of tendon, and girder length, averaging 49 girders, 194 anchors, and 2716 strands. Normality test showed that the measured prestress force per anchor head was normally distributed. The mean of the normal distribution could be obtained by dividing the total prestress force applied by the hydraulic jack by the number of strands inserted in the anchor head. The evaluation of the factors influencing the standard deviation revealed its dependency on the level of prestress only, and that it is not significantly affected by the length of the structure, tendon sag ratio, or number of strands. This evaluation enabled us to propose a formula estimating the standard deviation in terms of the mean prestress force so as to completely define the normal distribution. The so-obtained distribution of the prestress force, together with the measurement of the limited number of smart strands included in the anchor, were then used to track the change in the distribution of the prestress force in the in-service PSC structure. 

## Figures and Tables

**Table 1:** Mean and standard deviation of prestress force per anchor head based on structural factors of considered PSC structures.

No.	Girder Length (m)	Tendon Sag Ratio	Number of Strands in Tendon	Number of Measured Tendons	Mean of Mean Prestress Force per Anchor Head (kN)	Mean of Std. Deviation of Prestress Forces per Anchor Head (kN)
1	44.7	0.1082	13	2	196.4	5.9
2	44.7	0.0868	13	2	197.9	5.1
3	44.7	0.0546	13	2	198.3	5.2
4	44.7	0.0224	13	2	196.5	5.8
5	39.8	0.0974	13	3	199.5	8.4
6	39.8	0.0614	13	3	199.7	5.4
7	39.8	0.0252	13	3	199.8	7.4
8	42.4	0.1392	16	21	163.9	5.2
9	42.4	0.0966	16	21	163.7	5.3
10	42.4	0.0542	16	21	164.0	5.2
11	42.4	0.0118	16	22	163.8	5.5
12	44.9	0.1314	16	8	179.8	6.8
13	44.9	0.0914	16	8	179.3	7.0
14	44.9	0.0512	16	7	180.3	6.3
15	44.9	0.0112	16	7	179.9	7.0
16	49.3	0.1420	13	7	185.5	5.9
17	49.3	0.1144	13	7	185.2	6.2
18	49.3	0.0868	13	7	185.4	5.4
19	49.3	0.0592	13	6	185.2	6.2
20	49.3	0.0186	14	7	184.6	6.3
21	54.8	0.1278	14	7	183.2	5.8
22	54.8	0.1030	14	7	183.6	5.3
23	54.8	0.0782	14	7	184.4	5.5
24	54.8	0.0532	14	7	183.5	6.5
Total number of measured tendons				194		

**Table 2:** Hydraulic jacks used for the prestressing of girders and measurement of the prestress force distribution.

Hydraulic Jacks	Capacity (kN)	Stroke (mm)	Number of Holes
Company A	3000	220	13
Company B	4204	250	15
Company C	4500	300	19
